# Factors Associated with Free Medicine Use in Patients with Hypertension and Diabetes: A 4-Year Longitudinal Study on Full Coverage Policy for Essential Medicines in Taizhou, China

**DOI:** 10.3390/ijerph182211966

**Published:** 2021-11-15

**Authors:** Zhigang Guo, Lin Bai, Zhenhuan Luo, Mengyuan Fu, Liguang Zheng, Xiaodong Guan, Luwen Shi

**Affiliations:** 1 Department of Pharmacy, Peking University School and Hospital of Stomatology, Beijing 100081, China; guozhigang0909@163.com (Z.G.); zhenglg1103@163.com (L.Z.); 2 International Research Center for Medicinal Administration, Peking University, Beijing 100191, China; shiluwen211@163.com; 3 School of Pharmaceutical Sciences, Peking University, Beijing 100191, China; bailin@pku.edu.cn (L.B.); lawww@pku.edu.cn (Z.L.); mengyuan_fu@pku.edu.cn (M.F.)

**Keywords:** free medicine use, full coverage policy, hypertension, diabetes, essential medicines, China

## Abstract

Full coverage policies for medicines have been implemented worldwide to alleviate medicine cost burden and promote access to medicines. However, few studies have explored the factors associated with free medicine use in patients with chronic diseases. This study aimed to analyze the utilization of free medicines by patients with hypertension and diabetes after the implementation of the full coverage policy for essential medicines (FCPEM) in Taizhou, China, and to explore the factors associated with free medicine use. We conducted a descriptive analysis of characteristics of patients with and without free medicine use and performed a panel logit model to examine factors associated with free medicine use, based on an electronic health record database in Taizhou from the baseline year (12 months in priori) to three years after FCPEM implementation. After FCPEM implementation, the proportion of patients without any free medicine use decreased from 31.1% in the baseline year to 28.9% in the third year, while that of patients taking free medicines rose from 11.0% to 22.8%. Patients with lower income or education level, those with agricultural *hukou*, patients aged 65 and above, married patients, and patients in the Huangyan district were more likely to take free medicines. In conclusion, FCPEM contributed to improved medicine access, especially in vulnerable populations. Local policy makers should consider expanding the coverage of FCPEM to other types of medicines and cultivate the potential of social supports for patients to enhance the effectiveness of FCPEM policies.

## 1. Introduction

Patients with chronic diseases such as hypertension and diabetes experience heavy financial burden from medicine costs. Healthcare expenditures of diabetes, 90% of which were medicine costs, account for 12% of total healthcare spending [1,2]. Indeed, medicine cost burden is one of the key factors limiting access and adherence to medicines of patients with chronic diseases, especially in vulnerable populations such as low-income earners [3]. A literature review including 160 articles showed a significant association between increasing medicine cost and declines in medication adherence [4]. Limited access and adherence to medicines can compromise patient health, as good adherence is associated with a 19% reduction in the risk of cardiovascular disease and a 29% reduction in the risk of all-cause mortality [3,5]. They can also be costly to hospitals and the healthcare system through the increased use of avoidable healthcare services [6,7,8]. A report of the Organization for Economic Co-operation and Development estimated that every extra USD spent on medications for patients who adhere to medication can save between 3 to 13 USD on avoidable hospitalizations and emergency care [7]. To reduce medicine costs and promote access to medicines, the full coverage policy for medicines (FCPM) has been implemented in different forms, such as offering medicines free-of-charge or with copayment exemption around the world, especially for essential medicines [9,10,11,12,13].

China has implemented the full coverage policy for essential medicines (FCPEM) for antihypertensive and hypoglycemic medicines to promote medicine access and strengthen the management of hypertension and diabetes. Taizhou city of Zhejiang Province was among the first areas to pilot FCPEM. Between 2012 and 2013, each district in Taizhou selected medicines from the National Essential Medicines List of China (version 2012) and established its lists of free medicines with varying schedules. Patients using free medicines included in the regional list could waive medical-related costs at any primary health care institution or other designated institutions of the specific district. Physicians at these institutions were responsible for maintaining health records of patients, providing regular follow-ups, recording and reporting the medicines’ clinical benefits and appropriateness for the patient, and adjusting the medicine plan in a timely manner, such as quitting free medicines if clinical outcomes were poor. By October 2013, all districts of Taizhou had implemented their respective FCPEM policies.

Past evaluations of FCPM interventions for patients with chronic diseases proved effective in reducing costs, promoting medicine use and adherence, and preventing costly complications [14,15,16,17,18,19]. However, research also suggested that a considerable proportion of patients (8.9%) did not take or refill any medicines despite FCPM implementation [20]. Few studies have explored factors associated with free medicine use in patients with chronic diseases, which might affect the effects of FCPM on medicine use and adherence [17].

Filling this gap, this study aims to analyze the medicine utilization of patients with hypertension and diabetes after the implementation of FCPEM and to explore the factors associated with free medicine use.

## 2. Materials and Methods

### 2.1. Study Design

This was a retrospective study based on population-level pooled panel data in Taizhou, a prefecture-level city of Zhejiang Province with nine county-level or urban districts located in the central area of the Yangtze River Delta in China [21]. As the implementation schedule of FCPEMs differed among districts, we defined the baseline-year data as the records of the previous 12 months before FCPEM implementation and three-year follow-up data as the records of the 1–12, 13–24 and 25–36 months after FCPEM implementation. We subsequently generated a 4-year longitudinal sample. We then conducted a descriptive analysis and variance analysis of characteristics of patients with and without free medicine use and performed a logistic panel regression analysis to examine the variables that could be associated with free medicine use.

### 2.2. Data Source and Study Population

This study was based on a sample of observational data between 2011 and 2016 from the electronic health record database in Taizhou. Our analyses were limited to patients of the three districts (Huangyan, Linhai, and Wenling) as data of the other six districts was inaccessible due to systems upgrade. We extracted all electronic health records of patients with hypertension and diabetes in the three districts from the baseline year to three years after policy implementation. The records contained detailed information on demographics and health service utilization, including patients’ sex, age, *hukou*, marital status, educational level, type of insurance scheme, household income level, body mass index (BMI), smoking and drinking status, residence district, and medicine utilization [22].

**Figure 1:**
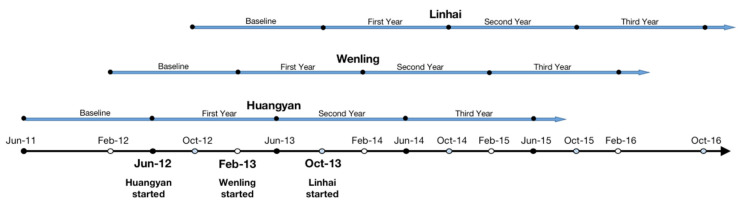
Study timeline of the full coverage policy for essential medicines in three districts in Taizhou, China.

The implementation schedule of FCPEM and medicines offered free-of-charge for each district are shown in Table S1, and the study timeline is presented in Figure 1.

### 2.3. Statistical Analysis

We conducted a descriptive analysis of the number and proportion of patients with and without free medicine use and compared the characteristics between the two groups through analysis of variance. We then performed a fixed-effects panel logit model that accounted for all stable potential confounders to examine the association of variables with free medicine use in sample patients after FCEMP implementation [23]. The calculation formula was:

logit Y = β_0_ + β × X + ε

where Y denoted free medicine use of patients, in which Y = 0 if the patient had not taken free medicines and Y = 1 if the patient took at least one free medicine during the study period; X indicated potential factors associated with free medicine use, including sex, age, *hukou*, marital status, educational level, type of insurance scheme, household monthly income per person, BMI, smoking status, drinking status, and residence district.

All statistical analyses were performed using STATA 14.0 (StataCorp LLC, College Station, TX, USA). Statistical significance was set at 2-tailed *p* < 0.05.

## 3. Results

A total of 271,951 patients with hypertension and diabetes were included at baseline, 318,654 were included in the first follow-up year, 340,238 in the second year, and 340,638 in the third year. As shown in Table 1, 86,971 (32.0%) of the total patients in the baseline year did not use any free medicines, while the proportion of patients without any medicine use decreased from 31.1% in the first follow-up year to 28.9% in the third year after FCPEM implementation. Among all patients, 35,042 (11.0%) used at least one free medicine in the first follow-up year (27.7% in Huangyan, 38.3% in Linhai, and 34.0% in Wenling), 59,973 (17.6%) in the second year (22.0% in Huangyan, 39.0% in Linhai, and 39.0% in Wenling), and 77,694 (22.8%) in the third year (19.3% in Huangyan, 35.7% in Linhai, and 45.0% in Wenling).

Table 2 shows the characteristics of the sample patients with and without free medicine use at the three-year follow-up. In the first year after FCPEM implementation, the proportions of patients with a household monthly income per person of less than 500 CNY (11.4% vs. 9.2%, *p* < 0.001), patients who were illiterate and semiliterate (44.4% vs. 40.9%, *p* < 0.001), patients with agricultural hukou (96.2% vs. 93.2%, *p* < 0.001), and patients aged 65 and older (55.0% vs. 50.7%, *p* < 0.001) in the sample patients with free medicine use were significantly greater than those in patients without free medicine use, and the same result was found for the second and third years.

The results from the logistic regression analysis are presented in Table 3. Patients with a household monthly income per person of less than 500 CNY were more likely to take free medicines than patients with a higher income (500–3000 CNY: β = −0.133, 95% CI = −0.217~−0.049, *p* = 0.002; >3000 CNY: β = −0.139, 95% CI = −0.225~−0.052, *p* = 0.002). Compared with patients with a highest educational attainment of middle school and above, illiterate and semiliterate patients were significantly more likely to take free medicines (middle school: β = −0.291, 95% CI = −0.368~−0.215, *p* < 0.001; high school and above: β = −0.975, 95% CI = −1.138~−0.812, *p* < 0.001). Patients with an agricultural *hukou* were more likely to take free medicines than those with a non-agricultural *hukou* (β = 0.793, 95% CI = 0.651~0.935, *p* < 0.001). In addition, patients aged 65 and above (β = 0.549, 95% CI= 0.502~0.596, *p* < 0.001) and married patients (β = 0.076, 95% CI = 0.012~0.140, *p* = 0.020) were more likely to take free medicines than their respective counterparts. Compared with patients in Huangyan, patients in Linhai (β = −1.135, 95% CI = −1.202~−1.067, *p* < 0.001) and Wenling (β = −0.492, 95% CI = −0.557~−0.427, *p* < 0.001) were significantly less likely to take free medicines.

## 4. Discussion

This study found an increase from 10.6% to 19.6% in the proportion of patients with free medicine use among patients with hypertension and diabetes at the three-year follow-up after FCPEM implementation and identified that low income, poor education, agricultural *hukou*, and advanced age were associated with free medicine use. Similar to previous studies conducted in other countries such as Canada and Brazil, we found that FCPEM implementation could improve medicine accessibility [15,24]. In various settings, improved medicine accessibility through FCPEM programs proved to help reduce patients’ direct medical costs and possibly lead to increased patient participation, continuity of treatment, and better health outcomes [15,24,25,26,27].

Furthermore, we found that patients with characteristics indicative of free medicine use in our study coincided with patients who had chronic illnesses and poor medicine use and adherence in previous studies [28,29,30]. This implies that FCPEM implementation can promote equitable access to medicines and improve the health outcomes of vulnerable populations. In addition, our results echo other studies that showed married patients were more likely to take free medicines than patients who were single [31,32], which might be explained by family support being promotive of medication adherence. It has been suggested that social support would help patients deal with the stresses of living with illnesses, leading to improved adherence to chronic illness treatment [32,33,34], which can be cultivated to improve future FCPEM implementation.

The selection of free medicines might have influenced the effect of regional free medicine policies. Our findings showed an imbalanced distribution of free medicine use among patients residing in different districts. Compared with patients in Huangyan, patients in Linhai and Wenling were less likely to take free medicines, which could be explained by the differences in the selection of medicines. A study in India showed that how well the list of free medicines matched with patients’ actual needs in a region had a critical impact on the actual policy implementation [17]. As merely a few medicines for hypertension and diabetes were selected to be provided free of charge in each district, many patients on other medicines could not benefit from the FCPEM policy. The selection of free medicines differed among the three districts included in our study and hence met the divergent medicine needs of patients, resulting in differences in matching. It might be necessary to reasonably expand or adjust the coverage of FCPEM. Moreover, patients can be further benefited by a provincial or national FCPEM plan to reduce regional differences in access to medicines [35].

Our study had several limitations. First, the database collected observational data on the collection and refills of medicines by patients, which might have introduced recall bias and other participant biases into our results. Second, data regarding six districts in Taizhou were unavailable during the study period due to system upgrades; we consequently limited our analyses to patients with hypertension and diabetes in the remaining three districts, which might affect the generalizability of our results. However, as the six districts were not excluded from analyses based on arbitrary criteria, and as we extracted all patient records from the sample districts, our sample was still representative of the general population in Taizhou to some extent. Third, we only included variables that had been collected in our database, and there might be other potential factors influencing of free medicine use, such as patients’ occupation. Fourth, it could be interesting and valuable to carry out further research on the association between patient medication needs and choices, determined by the patients’ disease status, detailed prescriptions of the patients, and other factors. Further research is needed to explore whether FCPEM implementation could improve medication adherence and clinical outcomes.

## 5. Conclusions

We found that FCPEM improved access to medicines for patients with hypertension and diabetes. Vulnerable populations such as patients with low income, poor education, agricultural *hukou*, and advanced age were more likely to benefit from FCPEM implementation. FCPEM could possibly contribute to equitable access to medicines and health outcomes. To enhance the effectiveness of FCPEM implementation, local policy makers should consider expanding the coverage of FCPEM to other types of medicines and cultivate social support from family members and the community of patients to realize the full potential of FCPEM.

## Figures and Tables

**Table 1:** Number and proportion of patients with and without free medicine use in Taizhou.

		No. (%) of Patients in Sample Districts			
		Baseline	First Year	Second Year	Third Year
**Overall**		271,951 (100.0%)	318,654 (100.0%)	340,238 (100.0%)	340,638 (100.0%)
**Patients without free medicine use**		271,951 (100.0%)	283,612 (89.0%)	280,265 (82.4%)	262,944 (77.2%)
	Patients without any medicine use	86,971 (32.0%)	99,122 (31.1%)	100,558 (29.6%)	98,498 (28.9%)
	Patients with medicine use excluding free medicines	184,980 (68.0%)	184,490 (57.9%)	179,707 (52.8%)	164,446 (48.3%)
**Patients with free medicine use**		/	35,042 (11.0%)	59,973 (17.6%)	77,694 (22.8%)
	Patients who quit taking free medicines	/	1232 (0.4%)	4205 (1.2%)	10,795 (3.2%)

**Table 2:** Characteristics of sample patients with and without free medicine use.

Characteristics		First Year			Second Year			Third Year		
		Without Free Medicine Use (*n* = 283,612)	With Free Medicine Use (*n* = 35,042)	*p*	Without Free Medicine Use (*n* = 280,265)	With Free Medicine Use (*n* = 59,973)	*p*	Without Free Medicine Use (*n* = 262,944)	With Free Medicine Use (*n* = 77,694)	*p*
Sex				0.000			0.000			0.000
	Female	58.5%	62.9%		58.3%	61.8%		58.3%	62.4%	
	Male	41.5%	37.1%		41.7%	38.2%		41.7%	37.6%	
Age				0.000			0.000			0.000
	<65	49.3%	45.0%		48.2%	43.8%		46.4%	42.7%	
	≥65	50.7%	55.0%		51.8%	56.2%		53.6%	57.3%	
Hukou				0.000			0.000			0.000
	Nonagricultural	6.8%	3.8%		7.0%	4.4%		7.1%	4.5%	
	Agricultural	93.2%	96.2%		93.0%	95.6%		92.9%	95.5%	
Marital status				0.000			0.000			0.000
	Single	16.7%	18.7%		15.8%	17.6%		15.2%	17.0%	
	Married	83.3%	81.3%		84.2%	82.4%		84.8%	83.0%	
Education				0.000			0.000			0.000
	Illiterate and semiliterate	40.9%	44.4%		39.2%	43.1%		38.1%	42.3%	
	Primary school	41.4%	41.4%		41.9%	42.3%		42.3%	42.9%	
	Middle school	14.5%	12.3%		15.4%	12.6%		16.0%	12.8%	
	High school and above	3.2%	1.9%		3.5%	2.0%		3.6%	2.0%	
Health insurance scheme ^a^				0.000			0.000			0.000
	None	12.2%	9.8%		12.4%	10.5%		11.9%	12.2%	
	URRBMI	85.5%	88.8%		85.1%	88.2%		85.4%	86.6%	
	UEBMI and CMI	2.3%	1.5%		2.5%	1.3%		2.7%	1.2%	
Household monthly income per person, CNY ^b^				0.000			0.000			0.000
	<500	9.2%	11.4%		8.6%	10.6%		8.2%	10.0%	
	500–3000	41.9%	50.8%		42.5%	47.1%		43.8%	44.5%	
	>3000	48.9%	37.8%		49.0%	42.3%		48.0%	45.5%	
BMI ^c^, kg/m^2^				0.000			0.000			0.000
	≤24	58.8%	57.5%		56.3%	54.9%		53.4%	50.6%	
	>24	41.2%	42.5%		43.7%	45.1%		46.6%	49.4%	
Smoking				0.444			0.002			0.000
	No	85.8%	85.5%		84.9%	85.4%		84.2%	85.6%	
	Yes	14.2%	14.5%		15.1%	14.6%		15.8%	14.4%	
Drinking				0.000			0.000			0.000
	No	93.0%	93.7%		92.5%	93.0%		91.3%	92.5%	
	Yes	7.0%	6.3%		7.5%	7.0%		8.7%	7.5%	
District				0.000			0.000			0.000
	Huangyan	14.8%	27.7%		16.1%	22.0%		17.9%	19.3%	
	Linhai	43.7%	38.3%		42.9%	39.0%		43.4%	35.7%	
	Wenling	41.4%	34.0%		41.0%	39.0%		38.7%	45.0%	

^a^ Health insurance scheme: URRBMI, Urban Rural Resident Basic Medical Insurance; UEBMI, Urban Employee Basic Medical Insurance (including free medical service); CMI, Commercial Medical Insurance. ^b^ CNY, Chinese yuan. ^c^ BMI, body mass index.

**Table 3:** Number and proportion of patients with and without free medicine use in Taizhou.

Associated Factors	β	*p* Value	95% CI	
			Inf	Sup
Sex (Ref: Female)				
Male	−0.298	<0.001	−0.352	−0.245
Age (Ref: <65)				
≥65	0.549	<0.001	0.502	0.596
Hukou (Ref: Nonagricultural)				
Agricultural	0.793	<0.001	0.651	0.935
Marital status (Ref: Single)				
Married	0.076	0.020	0.012	0.140
Education (Ref: Illiterate and semiliterate)				
Primary school	−0.045	0.099	−0.098	0.008
Middle school	−0.291	<0.001	−0.368	−0.215
High school and above	−0.975	<0.001	−1.138	−0.812
Health insurance scheme ^a^ (Ref: None)				
URRBMI	0.173	<0.001	0.090	0.256
UEBMI and CMI	−0.707	<0.001	−0.901	−0.512
Household monthly income per person, CNY ^b^ (Ref: <500)				
500–3000	−0.133	0.002	−0.217	−0.049
>3000	−0.139	0.002	−0.225	−0.052
BMI ^c^, kg/m2 (Ref: ≤24)				
>24	0.427	<0.001	0.388	0.466
Smoking (Ref: No)				
Yes	0.029	0.367	−0.034	0.091
Drinking (Ref: No)				
Yes	0.064	0.087	−0.009	0.138
District (Ref: Huangyan)				
Linhai	−1.135	<0.001	−1.202	−1.067
Wenling	−0.492	<0.001	−0.557	−0.427

^a^ Health insurance scheme: URRBMI, Urban Rural Resident Basic Medical Insurance; UEBMI, Urban Employee Basic Medical Insurance (including free medical service); CMI, Commercial Medical Insurance. ^b^ CNY, Chinese yuan. ^c^ BMI, body mass index.

## Data Availability

The data used in the study were mainly electronic health records and are not public and belong to the Health Department of Taizhou city in Zhejiang Province, which contains personal patient information. Other researchers may need to obtain permission from the Health Department of Taizhou city to access the data.

## Supplementary Materials

The following are available online at https://www.mdpi.com/article/10.3390/ijerph182211966/s1, Table S1: The implementation time and free medicines in the FCPEM.
