# Supplementary material for: Factors Associated with Free Medicine Use in Patients with Hypertension and Diabetes: A 4-Year Longitudinal Study on Full Coverage Policy for Essential Medicines in Taizhou, China
Source: Int J Environ Res Public Health. 2021 Nov 15;18(22):11966. doi: 10.3390/ijerph182211966 (PMC8620273; doi:10.3390/ijerph182211966)
Supplement: Supplementary file 1 [file ijerph-18-11966-s001.zip › ijerph-1411378-supplementary.pdf]

Table S1. The implementation time and free medicines in the FCPEMs.

District	Implementation Time	Antihypertensive Medicines Offered Free of Charge	Hypoglycemic Medicines Offered Free of Charge
Huangyan	June 2012	Captopril tablets, Indapamide tablets	Metformin Hydrochloride tablets, Glipizide tablets
	April 2013	Supplementary: Telmisartan tablets, Nitrendipine tablets, Compound Reserpine tablets, Zhenju antihypertensive tablets	Supplementary: Gliclazide tablets
Linhai	October 2013	Captopril tablets, Compound Reserpine tablets, Indapamide tablets, Nifedipine tablets	Metformin Hydrochloride tablets, Glipizide tablets
Wenling	February 2013	Captopril tablets, Compound Reserpine tablets, Hydrochlorothiazide tablets, Amlodipine Besylate tablets	Glipizide tablets, Metformin Hydrochloride Enteric-coated tablets
